# Analysis of Elimination Effects of Inbreeding on Genotype Frequency in Larval Stages of Chinese Shrimp

**DOI:** 10.3390/biology13040268

**Published:** 2024-04-17

**Authors:** Qiang Fu, Jingxin Zhou, Sheng Luan, Ping Dai, Ding Lyu, Baolong Chen, Kun Luo, Jie Kong, Xianhong Meng

**Affiliations:** 1 State Key Laboratory of Mariculture Biobreeding and Sustainable Goods, Yellow Sea Fisheries Research Institute, Chinese Academy of Fishery Sciences, Qingdao 266071, China; oucfuq@163.com (Q.F.); meditationzhou@163.com (J.Z.); luansheng@ysfri.ac.cn (S.L.); daiping@ysfri.ac.cn (P.D.); lvding@ysfri.ac.cn (D.L.); chenbl@ysfri.ac.cn (B.C.); luokun@ysfri.ac.cn (K.L.); kongjie@ysfri.ac.cn (J.K.); 2 Laboratory for Marine Fisheries Science and Food Production Processes, Qingdao Marine Science and Technology Center, Qingdao 266237, China

**Keywords:** *Fenneropenaeus chinensis*, inbreeding, larval development, segregation distortion, SNPs

## Abstract

**Simple Summary:**

In this research, we established inbred families of *Fenneropenaeus chinensis* with an inbreeding coefficient of 0.25, and studied how inbreeding depression affects the larval development. We examined gene frequency changes in two developmental stages through sequencing. The results showed significant inbreeding depression in larval survival rates during hatching and two major developmental stages, ranging from 24.36% to 45.28%. Sequencing at these stages revealed deviations from Mendelian segregation, with homozygote deficiency and heterozygote excess being the primary selection types. The elimination during the larval stage resulted in more heterozygotes being retained and increased the population’s heterozygosity. These findings indicate the presence of large amount of harmful recessive alleles in the Chinese shrimp genome and their potential criteria for elimination. The study provide valuable insights into the mechanisms of inbreeding depression in marine animals and provides guidance for shrimp population breeding strategies.

**Abstract:**

Marine animals possess genomes of considerable complexity and heterozygosity. Their unique reproductive system, characterized by high fecundity and substantial early mortality rates, increases the risk of inbreeding, potentially leading to severe inbreeding depression during various larval developmental stages. In this study, we established a set of inbred families of *Fenneropenaeus chinensis*, with an inbreeding coefficient of 0.25, and investigated elimination patterns and the manifestations of inbreeding depression during major larval developmental stages. Reduced-representation genome sequencing was utilized to explore the genotype frequency characteristics across two typical elimination stages. The results revealed notable mortality in hatching and metamorphosis into mysis and post-larvae stages. Inbreeding depression was also evident during these developmental stages, with depression rates of 24.36%, 29.23%, and 45.28%. Segregation analysis of SNPs indicated an important role of gametic selection before hatching, accounting for 45.95% of deviation in the zoea stage. During the zygotic selection phase of larval development, homozygote deficiency and heterozygote excess were the main selection types. Summation of the two types explained 82.31% and 89.91% of zygotic selection in the mysis and post-larvae stage, respectively. The overall distortion ratio decreased from 22.37% to 12.86% in the late developmental stage. A total of 783 loci were identified through selective sweep analysis. We also found the types of distortion at the same locus could change after the post-larvae stage. The predominant shifts included a transition of gametic selection toward normal segregation and other forms of distortion to heterozygous excess. This may be attributed to high-intensity selection on deleterious alleles and genetic hitchhiking effects. Following larval elimination, a greater proportion of heterozygous individuals were preserved. We detected an increase in genetic diversity parameters such as expected heterozygosity, observed heterozygosity, and polymorphic information content in the post-larvae stage. These findings suggest the presence of numerous recessive deleterious alleles and their linkage and suggest a major role of the partial dominance hypothesis. The results provide valuable insights into the mechanisms of inbreeding depression in marine animals and offer guidance for formulating breeding strategies in shrimp populations.

## 1. Introduction

Inbreeding is the mating of individuals with shared ancestry and can lead to a decrease in genetic diversity and the subsequent depression of certain phenotypic traits within a population [1,2]. Inbreeding depression is a matter of great concern across various species and holds significant implications for evolutionary processes, species conservation, and genetic breeding programs [3,4,5]. The genetic mechanisms of inbreeding depression have long been a subject of debate, with two major hypotheses proposed. The partial dominance hypothesis suggests that detrimental effects arise from the expression of recessive mutant alleles, whereas the overdominance hypothesis posits that heterozygotes possess a selective advantage [6]. Although the importance of these hypotheses remains a topic of discussion, more evidence supports the view that inbreeding depression is predominantly caused by the cumulative effects of deleterious mutations at multiple genetic loci [7].

Empirical evidence consistently indicates that inbreeding primarily impacts fitness-related traits rather than morphological traits [2]. However, the identification of inbreeding depression may fluctuate among studies due to various factors, like varying degrees of inbreeding, genetic diversity, trait types, developmental phases, and environmental factors [8]. The decline in fitness components caused by inbreeding can manifest at various stages of an organism’s life history, and lethal and sub-lethal mutations are more evident during early developmental stages, when severe depression is more readily detectable. Consequently, the study of inbreeding during the developmental stage yields more precise and meaningful results, since the early elimination of individuals allows for sampling a more heterogeneous pool of survivors, leading to lower estimates of inbreeding depression [8,9]. Marine animals possess genomes of significant complexity and heterozygosity. Moreover, their great fecundity combined with high larval mortality rates [10] increases the likelihood of inbreeding, potentially resulting in severe inbreeding depression during various larval developmental stages. These characteristics make marine animals ideal models for studying the effects of inbreeding.

Inbreeding depression has been widely reported in aquaculture species [9,11,12,13,14]. In shrimp, the impact of inbreeding on typical economic traits and the effect of varying levels of inbreeding have been extensively studied [9,15,16,17]. However, few studies have been devoted to the specific genetic loci involved and the underlying mechanisms responsible for the elimination effect, particularly during the larval developmental stages. The fundamental principle of inbreeding cannot be completely determined by analysis only at the phenotypic level. The molecular basis of inbreeding can be better understood by studies at the molecular level, which benefit from high-throughput sequencing. In our previous studies, we developed a novel classification method that can discern the selection of different genotypes in segregation distortion [18]. This method can also be applied to investigate the selection effects on genotype frequency during larval stages under inbreeding conditions. By employing this approach, we can gain valuable insights into the criteria for elimination and the genetic mechanisms involved at the molecular level.

*Fenneropenaeus chinensis* is one of the most valuable mariculture species in China. In 2005, a breeding program was initiated with the aim of improving its growth rate and disease resistance. The breeding strategy was based on large-scale family selection. Despite careful control of inbreeding (<1% per generation), the closed breeding population inevitably experienced growth in inbreeding. Performance traits exhibited inbreeding depression ranging from 4.16% to 4.74% per 10% increase in inbreeding within this population. However, traits related to overall fitness did not show significant decline during the juvenile and adult stages [15]. This inconsistency may be attributed to the early elimination of lethal and sub-lethal mutations during the larval stage, which could mask subsequent declines [8]. Nevertheless, further confirmation is still required.

The objective of this study was to investigate the selection characteristics of inbred families during the larval stages from the perspective of genotype frequency. The results offer insights into the genetic foundation of significant elimination during the larval stage of marine penaeid shrimp. Additionally, they can provide valuable guidance for the control of inbreeding within breeding programs for *F. chinensis*.

## 2. Materials and Methods

### 2.1. Research Population and Family Construction

The parental population for this study was derived from G14 of the core breeding population of *F. chinensis*, known as “Huanghai No. 5”. Its base population was constructed by crossing six different geographic populations in 2005 and has since been continuously selected using pedigree methods and large-scale family selection. The family construction and tests were conducted at the Marine Genetic Breeding Center of the Chinese Academy of Fishery Sciences in Qingdao, China. After promoting sexual maturity for 60 days, the prawns were mated through artificial insemination following a specific mating design. The control families were established by carefully mating known pedigree males and females based on their estimated breeding values, following optimal contribution selection criteria and with the inbreeding coefficient controlled within 1%. The experimental inbred lines were established by mating full-sibling individuals within the same family, with an inbreeding coefficient of 0.25. The experiment group included three sets of inbreeding families, and each of them comprised five full-sibling F_2_ families. For each set, five control families were also established, which were non-inbreeding mated while being half-sibling to the inbreeding families.

**Figure 1:**
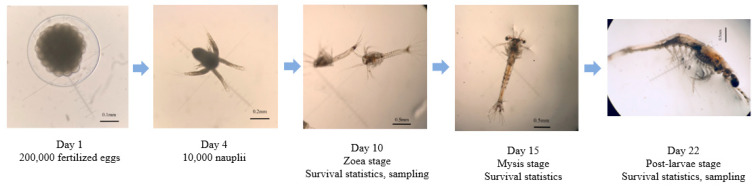
The infographic for larvae development stages.

After artificial insemination, the parent prawns were transferred to separate 200 L buckets for spawning. Seventy-two hours after fertilization, three 1 mL samples were taken from each family and observed under a microscope to determine the hatching rates. Subsequently, 10,000 nauplii for each family were randomly selected, transferred to larvae-culture buckets, and cultured according to a standardized procedure [19]. The larvae underwent Nauplius stage (N), Zoea stage (Z), Mysis stage (M) and Post-larvae stage (P) over approximately 21 days (Figure 1). At each developmental stage, three 100 mL samples were taken from each family bucket to calculate larval density and the number of survivors. Simultaneously, 100 larvae were randomly sampled from each developmental stage, fixed with 95% alcohol, and stored at −80 °C. At the P5 stage, all post-larvae were collected, and the overall survival rate during the entire larval rearing period was calculated. A total of 1500 larvae were retained and transported to new tanks for further rearing.

### 2.2. Sequencing Library Construction

Out of the 15 F_2_ families, the inbred family named 714Y, which exhibited a high mortality rate during larval development, was selected for further genetic analysis. We employed a reduced-representation sequencing strategy (RRS) to analyze the genotype frequency at two different developmental stages. For this purpose, 2b-RAD libraries were prepared for two parents and 36 larvae in zoea and post-larval stages (P3), respectively, following the protocol of Wang et al. [20]. Genomic DNA was extracted using the standard phenol/chloroform method. We constructed standard BsaXI libraries with original adaptors that covered nearly all recognition sites of this Type IIB restriction enzyme in the *F. chinensis* genome. Each library was ligated from multiple restriction enzyme-digested fragments and tagged with a unique barcode during preparation to simplify the pooling process [21]. After quality check, all libraries were subjected to an Illumina Hiseq Xten sequencer (Illumina, San Diego, CA, USA) for 2 × 150 bp paired-end sequencing.

### 2.3. Data Processing and Genotyping

The raw data were filtered by removing adaptors and reads of poor quality (those with over 15% of bases having a quality value below Q30 or an N base proportion exceeding 8%). The remaining high-quality reads were then applied to de novo 2b-RAD genotyping using the RAD-typing program v1.0 under optimized default parameters for marine animals [22]. A reference of parent-shared representative sites was established based on the male and female parent database and ustacks v2.1.3 software [23], which was utilized for subsequent locus genotyping. High-quality reads from all larvae were genotyped by aligning them to these reference sites and assessed using a likelihood ratio test (−r 0, −M 4, −v 2) [24]. Markers that were polymorphic and heterozygous in at least one parent and could be genotyped in at least 80% of the larvae were considered as qualified segregating markers. To ensure the accuracy of the analyses, genotyping results were further refined by excluding sites with a minor allele frequency (MAF) lower than 0.01, non-biallelic SNP markers, and sites with multiple SNPs within a tag. The resulting markers were retained for subsequent genotype frequency analysis.

### 2.4. Genotype Frequency Analysis

In each developmental stage, three types of polymorphic markers were observed. *hk* × *hk* represented markers where both parents were heterozygous, and *lm* × *ll* indicated markers where only the male parent was heterozygous. *nn* × *np* denoted markers where only the female parent was heterozygous. These markers were evaluated using the chi-square test to determine if they conformed to the Hardy–Weinberg equilibrium, which was expected to be 1:2:1, 1:1, and 1:1, respectively. Markers that deviated from the expected Mendelian ratio in the *hk* × *hk* type were subjected to further analysis. The gametic or zygotic selection types were determined by two successive chi-square tests (Table 1) following the separation detection model of F_2_ generation markers based on the maximum likelihood method [25].

**Figure 2:**
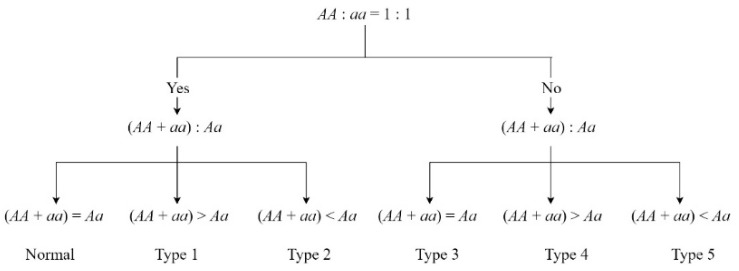
Five classifications of zygotic selection SNPs.

Regarding the zygotic selection markers, the eliminated genotypes were screened out by the ratio of three genotypes following the classification method we developed [18]. This involved evaluating the ratio between the two homozygotes in the segregating markers (*AA*:*aa*) and the ratio between homozygotes and heterozygotes ((*AA* + *aa*):*Aa*) using chi-square tests in succession (Figure 2). To better understand the severity of marker deviation, we transformed the *p*-value of each distorted marker into its natural logarithm (−Log10 *p*) to conform to a normal distribution. We refer to this normalized *p*-value as the Segregation Distortion Value (SDV). The higher the SDV, the lower the *p*-value became, and this indicated more severe segregation distortion at that locus.

### 2.5. Comparison of Genetic Parameters and Locus Frequencies

Genetic diversity parameters in the two developmental stages, including the effective number of alleles (*Ne*), expected heterozygosity (*He*), observed heterozygosity (*Ho*), polymorphic information content (*PIC*), nucleotide diversity (*P_i_*), and allele frequencies, were estimated using genepop (Version 1.0.5) [26]. Selective sweep analysis was performed to identify loci under selection from the zoea to the post-larval stage. This method combined the population differentiation coefficient (*F_ST_*) and the nucleotide diversity (*P_i_*) to identify strong signals of selection. Loci with a minimum allele frequency (MAF) below 0.05 were further filtered out using vcftools software v0.1.13 in both groups. For the remaining loci, the *F_ST_* and *P_i_* values were calculated. The selective sweep region was determined by selecting loci that fell within the top 5% highest *F_ST_* region, the bottom 5% *P_i_* region, and the top 5% *P_i_* region. Loci within this intersection region were considered as candidate loci.

### 2.6. Statistical Analysis

Analysis of variance (ANOVA) was performed to assess the survival rate at different developmental stages, and multiple comparisons were conducted using SPSS 27.0. The significance threshold was set at *p* < 0.05.

## 3. Results

### 3.1. Descriptive Statistics of Larval Survival

The hatching rates and survival rates at different larval developmental stages are presented in Table 2. The average hatching rate of the inbred families was significantly lower (24.36% lower) than that of the control families. After normalizing the density of hatched nauplii, the mortality of larvae from the nauplius to the zoea stage and after post-larval metamorphosis was relatively low in both control and inbred groups. However, substantial mortality occurred during the zoea to mysis stage and the mysis to post-larvae stage, with larval survival rates decreasing to 65% and 53% in the control families, respectively. Moreover, the survival performance of the inbred families during these two stages was worse. The inbreeding depression rate of larval survival was 29.23% and 45.28% in the mysis and post-larvae stages, respectively. The results indicated that the predominant larval elimination, as well as the effects of inbreeding depression, occurred during the hatching stages and metamorphosis stages of mysis and post-larvae.

### 3.2. Sequencing Data Processing and Marker Development

**Figure 3:**
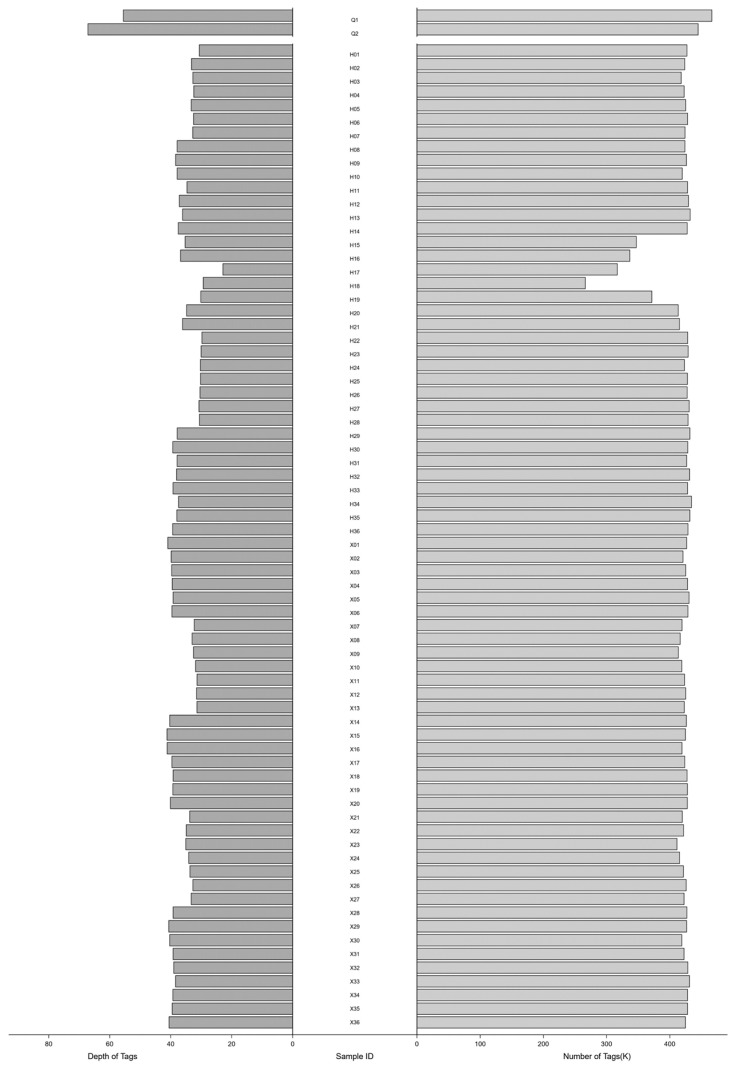
The number of unique tags from each library and their sequencing depth. The vertical axis represents the identification numbers of 74 samples, Q represents the parent sample, H represents the zoea stage, and X represents the post-larvae stage.

In total, we obtained 63.11 Gb of clean sequencing data from 74 samples. The average number of reads for the two parents was 67.71 million, and the high-quality reads accounted for 65.08%. As for the 72 larval samples, the average number of sequencing reads was 30.58 million, and the percentage of high-quality reads was 77.05%. The reference tag database, clustering reads from the two parents, included a total of 522,402 tags, which served as a reliable reference for the subsequent genotyping. After aligning the high-quality tags of each larval sample to this reference, we obtained an average of 418,145 unique tags per sample, with an average mapping rate of 63.41%. The sequencing depth for the two parents was 55.56× and 67.15×, respectively, while for the larvae, it ranged from 31.36× to 41.22×, with an average depth of 35.68×. The number of unique tags from each library and their sequencing depth are shown in Figure 3. A total of 31,767 polymorphic SNPs were identified through the RAD-typing program. After filtering, there were 29,435 biallelic SNPs shared by the two developmental stages. These selected segregating markers were then subjected to further analysis.

### 3.3. Changes in Genetic Diversity between the Two Stages

Out of all 29,435 segregating markers, the Ts/Tv ratio was 1.38 in all larval samples. Using the major allele at each locus during the zoea stage as a wild type, the average frequency of major alleles at all loci was 75.33% in the zoea stage but decreased to 70.34% in the post-larvae stage. In comparison to the zoea stage, there were 15,306 loci in the post-larvae stage, where the frequency of the wild-type allele changed by more than 5%. Among these, 11,094 loci had a decrease in wild-type allele frequency, accounting for 37.69% of all loci; while 4212 loci had an increase in wild-type allele frequency, accounting for 14.31% of all loci. This seems to be a type of balancing selection, where heterozygotes are preferred during larval selection. From the perspective of genetic diversity parameters, observed heterozygosity was higher than expected heterozygosity in both stages, and both exhibited an increasing trend (Table 3). From the zoea to post-larvae stage, the expected heterozygosity increased from 0.36 to 0.38, and the observed heterozygosity increased from 0.47 to 0.50. Other genetic diversity parameters, such as the effective number of alleles, polymorphic information content, and nucleotide diversity, also showed increases.

**Figure 4:**
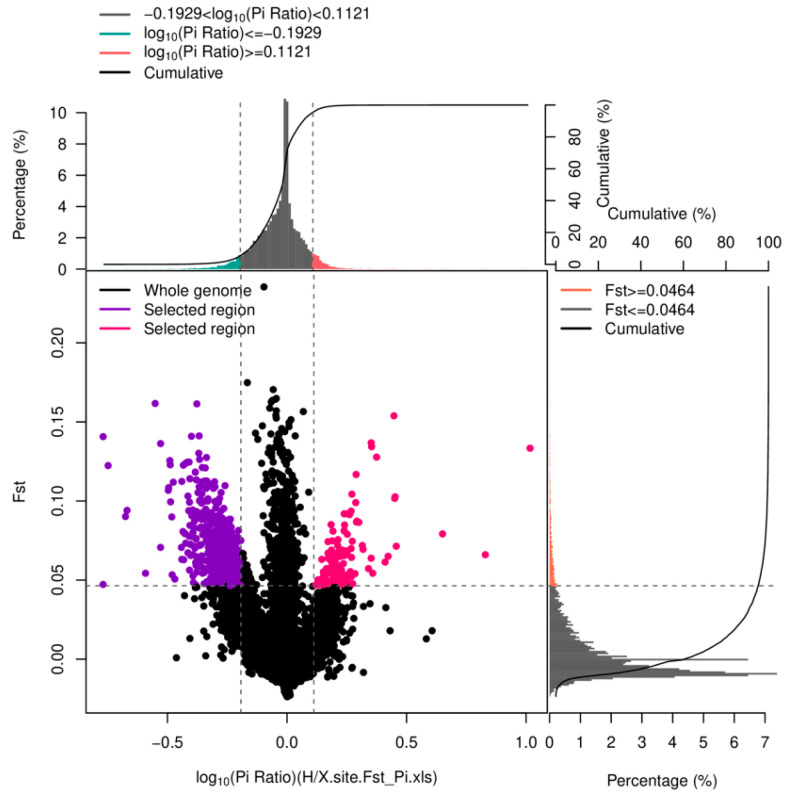
Selective sweep analysis of candidate loci under selection from zoea to post-larvae. The *x*-axis represents the Log10 (*P_i_* ratio) values, while the *y*-axis represents the *F_ST_* values, corresponding to the frequency distribution plot located above and to the right, respectively. The topmost green and red regions represent the bottom and top 5% regions selected based on *P_i_* values, while the orange region on the right indicates the top 5% regions selected based on *F_ST_* values. The overlapping red and purple region of the scatter plot in the middle represents the intersection of *F_ST_* and *P_i_* and is considered to represent the candidate loci.

The average *F_ST_* value estimated from the shared SNPs between the two stages was 0.0043, indicating a fundamental consistency in their genetic structure. A total of 783 loci were identified based on the *F_ST_* and *P_i_* values (Figure 4). These loci served as important candidates under selection from the zoea to post-larvae stages.

### 3.4. Overall Manifestation of Segregation Distortion in Larvae

Among the 29,435 common SNPs shared between the two stages, 22.37% exhibited significant deviation from the expected Mendelian segregation in the zoea stage, while the proportion of distorted markers decreased to 12.86% in the post-larvae stage (Table 4). Three types of segregating markers showed distinct proportions and degrees of distortion. The proportion of distorted markers was higher in the *hk* × *hk* type compared to markers with only one heterozygous parent. No significant difference was observed between the *ll* × *lm* and *nn* × *np* types. This trend was particularly evident in the post-larvae stage.

**Figure 5:**
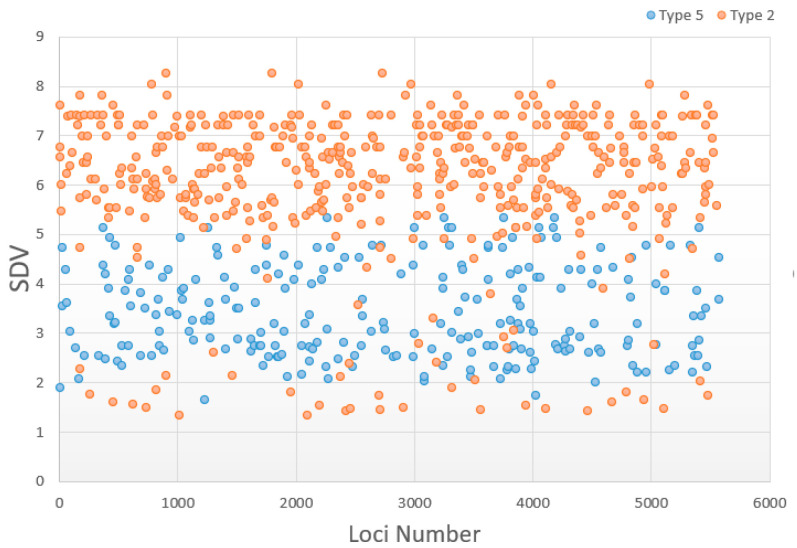
The SDR distribution of the two primary distorted markers in the zoea stage. The ordinate value refers to segregation distortion values (SDVs) of these markers. Type 2 and type 5 markers refer to those classifications of zygotic selection in Table 5.

The analysis of markers from the *hk* × *hk* type revealed that the proportion of gametic selection was 45.95% in the zoea stage and decreased to 29.28% in the post-larvae stage. Type 2 loci with heterozygote excess and type 5 loci with homozygote deficiency were the predominant types of zygotic selection in both stages. The sum of the two types accounted for 82.13% of zygotic selection in the zoea stage and 89.91% in the post-larvae stage (Table 5). In the zoea stage (Figure 5), the average SDR of type 2 markers was 6.07, significantly higher than the average SDR of 3.42 for type 5 markers (*p* < 0.001). The characteristic of SDR in the post-larvae stage was the same. These results indicated a greater selection pressure on loci with heterozygote advantage compared to those exhibiting homozygous deletion.

For single-parent heterozygous loci, the results from Table 4 indicate a noticeable decrease in their deviation ratios from the zoea stage to the post-larvae stage. The specific elimination types are presented in Table 6. In both stages, the heterozygotes rather than the homozygotic types were predominant in suffering from selection. Among all deviated markers, the proportions of the *lm* and *np* types under elimination were 94.16% and 92.99% in the zoea stage, respectively; in the post-larvae stage, these proportions decreased to 85.02% and 84.69% for the *lm* and *np* types, respectively. Mutant alleles of *m*/*p* possibly showed a disadvantage in gametes or zygotes and were more likely to be eliminated, and thus these loci were considered as dominant. This result further supported the partial dominance hypothesis.

### 3.5. Change in Distorted Types

**Figure 6:**
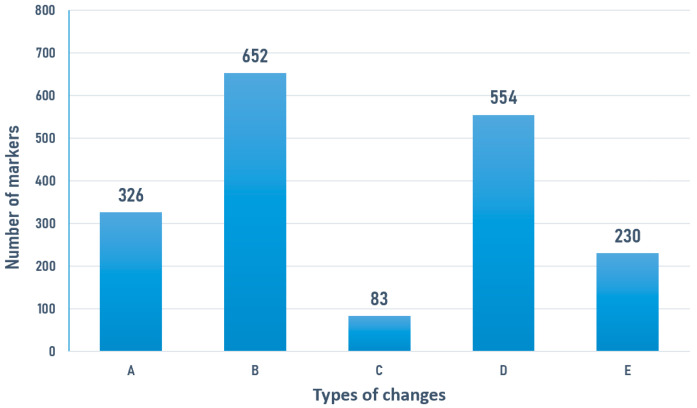
The main five types of changes in segregation distortion. A: normal segregation to distorted segregation; B: gametic selection to normal segregation; C: homozygous deficiency to heterozygous excess; D: other types of distortion to heterozygous excess; E: other types of distortion to homozygous deficiency.

We further compared the types of distorted loci between the two developmental stages and observed potential changes in the types of distortion at the same locus as the larvae were eliminated. Out of 5582 *hk* × *hk* parental heterozygous loci, 3589 loci showed normal segregation in both stages. The five main types of changes in distorted loci are shown in Figure 6. Among these, 326 loci shifted from normal segregation to distorted segregation, and 652 loci shifted from gametic selection to normal segregation. A total of 83 loci shifted from homozygous deficiency to heterozygous excess, and 554 loci shifted from other types of distortion to heterozygous excess. In addition, 230 loci shifted from other types of distortion to homozygous deficiency.

## 4. Discussion

Compared to terrestrial animals, marine animals have a distinct reproductive system, characterized by high fecundity, rapid development through a planktonic larval stage, and significant early mortality rates [27]. This adaptation enables them to survive in fluctuating ocean environments. Hedgecock [28] introduced the concept of Sweepstakes Reproductive Success (SRS) to describe this unique reproductive strategy, where only a few individuals succeed while many others do not. SRS is based on the idea of a wide variation in individual reproductive success during competitive life cycles [10], providing an opportunity for inbreeding effects to influence selection outcomes and potentially serve as a significant driving force for natural selection. In this study, we investigated the eliminated characteristics of the main larval developmental stage of Chinese shrimp and the effects of inbreeding. The results of genotype segregation at two substantial elimination stages indicated that the selection after inbreeding resulted in significant heterozygote excess and homozygous deficiency. More heterozygous individuals were retained due to the selection process.

It has been reported that natural elimination mainly occurred in the larval developmental stages in the life history of *F. chinensis*, and the inbreeding depression in mortality rates was not evident after the post-larvae stage [15]. Our study further revealed that elimination mainly occurred in hatching (from fertilized eggs to nauplius), the zoea to mysis period, and the mysis to post-larvae period (Table 2). The results of depression rates in the inbred group indicated that a decrease of larval mortality rates due to inbreeding was also primarily observed during these three stages. The depression rate (F = 0.25) of hatching as well as survival in the mysis and post-larvae stages was 24.36%, 29.23% and 45.28%, respectively. These levels were much higher than the depression of body weight observed in P140 (4.16% per 10% increase in F) [15] as well as in adult shrimp (2.19% per 10% increase in F) of *L. vannamei* [16]. In contrast, the decline in mortality rates for juvenile and adult shrimp was significantly lower than that during the larval stage, which was 1.77% and 0.009% per 10% increase in F in the above two studies. Similar results were also found in inbreeding studies of *E. carinicauda* [17]. This aligned with the empirical evidence that inbreeding depression is severe in traits that are closely related to fitness, such as infant survival [29], and was consistent with studies in other marine animals [30]. Based on the above, in the subsequent genotype frequency study, we focused on the zoea and post-larvae stages. These two stages can help us understand how the genotype frequency of each locus changes after the first major elimination and the three main elimination events.

Until the cost of resequencing becomes affordable for non-model organisms, the RRS technique remains an economical and efficient method for obtaining genotypes at specific loci. In this research, 2b-RAD was applied, since this technology featured even and tunable genome coverage in order to provide reliable and flexible large-scale SNPs [20]; it has successfully been applied to the construction of high-resolution linkage maps of *C. farreri* [22] and *F. chinensis* [31]. Sequencing produced an average of 67.71 and 35.68 million reads for parental and progeny libraries, respectively. After quality filtering, more than 65.08% of parental reads and 77.05% of progeny reads remained. Compared to our initial application of this technique in constructing the first high-density genetic map of *F. chinensis*, there was a decrease in the proportion of high-quality reads (85% in that research), possibly due to the use of isolength restriction site–associated DNA (isoRAD) [21] in this sequencing. However, due to a significant increase in sequencing output, the sequencing depth of the parents and progenies (61× and 36×) also significantly increased. This depth far exceeded the suggested depth (20× and 15× for parents and progenies through simulation analysis, with a genotyping accuracy > 96%) [24]. There was no difference in the high-quality reference assembled from the two parents. Therefore, we believe that the markers we developed were reliable.

Segregation distortion is a common occurrence in genetic studies. Deviations from Mendelian segregation ratios have been reported in various marine animals, including prawn [32,33,34]. Typically, only a few markers show deviation, and they are excluded from the genetic analysis. However, segregation distortion was an indication of the linkage between molecular markers and distorting factors such as recessive detrimental genes [35]. It can therefore be utilized to study the quantity of inbreeding loads as well as the selected alleles and genotypes. A few days after hatching, in the zoea stage, 54.05% of the parental heterozygous loci were of the zygotic selection type, while 45.95% resulted from gamete abortion. This suggested that both zygotic and gametic selection were present and had a significant impact on segregation distortion. Further analysis revealed that within the zygotic selection type SNPs, 27.30% exhibited homozygous deficiency and 54.83% showed heterozygous excess. This indicated that overdominance and partial dominance hypotheses both played a crucial role in explaining inbreeding depression. The above evidence points to the fact that deleterious recessive mutations are abundant in shrimps and they possibly play a major role in inbreeding depression.

In the two larval developmental stages, the overall segregation distortion ratios were 22.37% and 12.86%, respectively. These figures were similar to those reported in mapping populations of marine animals [33,34,36,37] but were lower than the 50.77% observed in another study on a Chinese shrimp family [18]. In that study, adult shrimps from a full-sibling family underwent artificial selection, with an intensity exceeding 3%. The high selection pressure could potentially explain the substantial severe deviation. Our findings also revealed a decrease in segregation distortion ratios from the initial discernible elimination to the third main elimination event. In addition, we observed a reduction in gametic segregation distortion ratios, decreasing from 45.95% in the zoea stage to 29.28% in the post-larval stage. Given that gametic selection was essentially completed before hatching, this decline suggested the potential selective elimination of another wild-type allele at the zygotic stage, if not due to sampling error. Due to constraints in experimental design and sequencing costs, we did not establish replicate groups in the sequencing of samples from the two developmental stages. This could potentially introduce some sampling errors into the statistical analysis of genotype frequencies. To minimize the risk of such errors, we did not select larvae samples based on size, with the aim of preserving their size distribution within a normal range.

These seemingly perplexing results can be accounted for by considering the pseudo-overdominance effect. The pseudo-overdominance effect, reviewed by Charlesworth and Willis [7], is primarily caused by selection on neutral loci due to the genetic hitchhiking effect [38,39]. We also found and elaborated on evidence of this effect in a separate study within the mapping population of Chinese shrimp [18]. The shrimp genome exhibits high heterozygosity and leads to the presence of numerous deleterious recessive alleles. Inbreeding depression thus manifests particularly severely in shrimp and the majority of marine animals [27]. Due to the presence of numerous deleterious loci and their linkage relationships, neutral loci might also undergo significant selection pressure because of their linkage with detrimental loci during ongoing elimination process in the larval stage. This could explain the reduced gametic and overall segregation distortion ratios. We speculated that, following the pseudo-overdominance effect, subsequent selection might target neutral homozygotes and lead to a decrease in the proportion of normal alleles, which was a form of balancing selection. In the comparison between two stages, we identified 783 loci through selective sweep analysis. These loci might potentially be neutral loci caused by genetic hitchhiking effects, so we did not conduct further gene functional and enrichment analysis. However, they at least verify that different loci may be under selection during different developmental stages. Under this selection pattern, the elimination effect of detrimental alleles at the gametic stage was weakened. The gametic selection loci could possibly become normally segregated. Supporting evidence could be seen in the change of distortion types between the two stages (Figure 6). There were 683 gametic selection loci whose distortion types changed in the post-larvae stage. The vast majority (95.46%) of them shifted to normal segregation loci. Additionally, 554 loci shifted from other types to heterozygote excess, indicating a reduction in homozygotes for both alleles due to continuous selection pressure. Notably, as observed in the results of single parental heterozygous selection loci (Table 6), the significant increase in the elimination rate of wild-type homozygotes *ll* and *nn* in the post-larval stage provided further supporting evidence to some extent.

The Ts/Tv ratio observed in our study was 1.38 in all larvae and was higher than that reported in other species. For instance, scallops [40] exhibited ratios ranging from 1.07 to 1.13, while fish [41] showed ratios ranging from 1.22 to 1.24. This may be attributed to the conversion deviation strategy proposed by Wakeley [42], which is a potential mechanism to counteract negative effects during long-term evolutionary processes. Our findings might be consistent with this concept, particularly considering the high reproductive capacity and elimination rate observed in crustaceans.

The impact of inbreeding on populations over the long term leads to an increase in the frequency of homozygous genotypes and a decrease in heterozygous frequencies. This is a phenomenon well-documented in most model organisms [7] and shrimps [17]. Our study focuses on the changes in genotype frequency influenced by inbreeding during the larval development stages in one generation. Through the analysis of genetic diversity parameters in two stages, it was observed that all five parameters calculated based on different genotype frequencies exhibited varying degrees of increase in the post-larval stage (Table 3), aligning with the decrease in segregation distortion ratios. This illustrated, from another perspective, that the selection process resulted in a higher proportion of heterozygotes, which supported a significant role of overdominance but could be readily explicable by deleterious mutations [7]. The above results also gave a possible interpretation of why it was difficult to explain the large effect of inbreeding on fitness in model species without a significant contribution from variability as maintained by selection [43], as well as why mutation due to deleterious alleles was not the sole source of the variation, which can only explain only about 60% of the observed inbred load for fitness components [44].

## 5. Conclusions

Noticeable purging selection and inbreeding depression occur in certain larval developmental stages of Chinese shrimp. Homozygote deficiency and heterozygote excess are the two major types of zygotic selection. Possibly owing to the genetic hitchhiking effect of deleterious loci, the distortion types of the same locus can change over different developmental stages. Nature selection tends to retain more heterozygotes after larval developmental stages. The results provide valuable insights into the mechanisms of inbreeding depression in marine animals, which possess genomes of considerable complexity and heterozygosity and reproductive characteristics with high elimination rates. It also offers guidance for formulating breeding strategies in marine economic animal and marine biodiversity conservation.

## Figures and Tables

**Table 1:** Selection type test of deviated *hk* × *hk* markers.

Test Formulae	Test of Significance		
$x_{1}^{2}=\frac{{\left(2np-n\right)}^{2}+{\left(2nq-n\right)}^{2}}{n}$	×	√	√
$x_{2}^{2}=\frac{{\left(n_{AA}-np^{2}\right)}^{2}}{np^{2}}+\frac{{\left(n_{Aa}-2npq\right)}^{2}}{2npq}+\frac{{\left(n_{aa}-nq^{2}\right)}^{2}}{nq^{2}}$	√	√	×
Selection type	Zygotic	Zygotic	Gametic

*p* = frequency of allele *A*; *q* = frequency of allele *a*; *n* = total larvae number that genotyped in each stage; *n_AA_*, *n_Aa_*, *n_aa_* represented the number of the three genotypes; √ means *p* < 0.05 and × means *p* > 0.05.

**Table 2:** Hatching rates and larval survival at different developmental stages.

	Hatching Rates	Larval Survival in Z	Larval Survival in M	Larval Survival in P3	Larval Survival in P10
Control families	0.78 ± 0.07 ^a^	0.91 ± 0.04 ^a^	0.65 ± 0.08 ^a^	0.53 ± 0.09 ^a^	0.48 ± 0.08 ^a^
Inbred families	0.59 ± 0.14 ^b^	0.89 ± 0.06 ^a^	0.46 ± 0.12 ^b^	0.29 ± 0.14 ^b^	0.27 ± 0.13 ^b^
Inbreeding depression	24.36%	2.20%	29.23%	45.28%	43.75%

Different superscript letters within columns indicate significant difference (*p* < 0.05).

**Table 3:** Genetic diversity parameters in the two developmental stages.

Developmental Stage	*He*	*Ho*	*PIC*	*Ne*	*P_i_*
Zoea	0.3641	0.4735	0.2935	1.6075	0.3703
Post-larvae	0.3826	0.5087	0.3060	1.6489	0.3886

*He*: expected heterozygosity; *Ho*: observed heterozygosity; *PIC*: polymorphic information content; *Ne*: effective number of alleles; *P_i_*: nucleotide diversity.

**Table 4:** Ratios of distorted markers in different types of segregating markers between the two developmental stages.

Marker	Type Specification	Number	Ratios of Distortion in Zoea	Ratios of Distortion in Post-Larvae
*hk* × *hk*	Biparental heterozygous	5582	27.73%	20.71%
*lm* × *ll*	Male heterozygous	11,793	21.06%	11.38%
*nn* × *np*	Female heterozygous	12,060	26.84%	10.67%
Total		29,435	22.37%	12.86%

**Table 5:** Classifications of zygotic selection SNPs in the two developmental stages.

Classification	Normal	Type 1	Type 2	Type 3	Type 4	Type 5
Number in zoea	3915	96	494	57	8	246
Ratio in zoea	-	10.65%	54.83%	6.33%	0.89%	27.30%
Number in post-larvae	4349	38	554	49	1	230
Ratio in post-larvae	-	4.36%	63.53%	5.62%	0.11%	26.38%
Possible explanation	Mendelian segregation	Heterozygote deficiency	Heterozygote excess	Impact of partial recessive deleterious		Homozygous deficiency

**Table 6:** Elimination type of single-parent heterozygous loci in the two developmental stages.

Type	Genotype under Elimination	Number/Ratio in Zoea	Number/Ratio in Post-Larvae
*lm* × *ll*	*lm*	2339/94.16%	1141/85.02%
	*ll*	145/5.84%	201/14.98%
*nn* × *np*	*np*	2373/92.99%	1090/84.69%
	*nn*	179/7.01%	197/15.31%

## Data Availability

The data presented in this study are available on request from the corresponding author due to the protection of breeding population information.
